# Supplementary material for: Smoking Suppresses the Therapeutic Potential of Adipose Stem Cells in Crohn’s Disease Patients through Epigenetic Changes
Source: Cells. 2023 Mar 27;12(7):1021. doi: 10.3390/cells12071021 (PMC10093550; doi:10.3390/cells12071021)
Supplement: Supplementary file 1 [file cells-12-01021-s001.zip › Supplementary Figures.pdf]

Figure S1.

A.

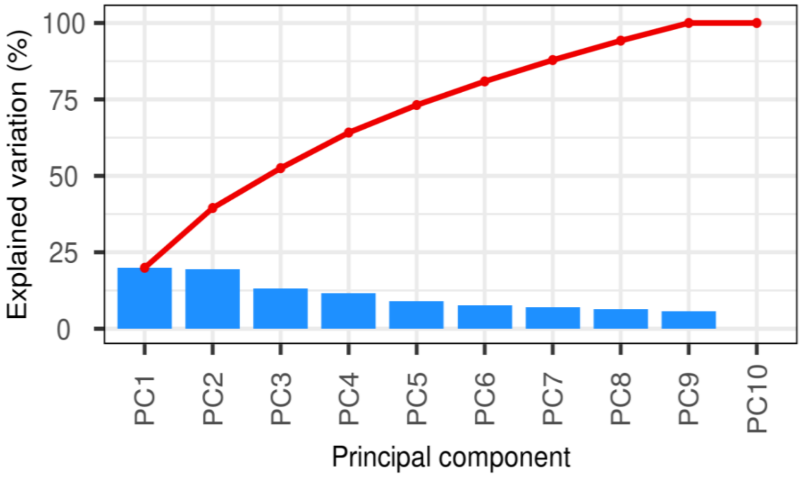

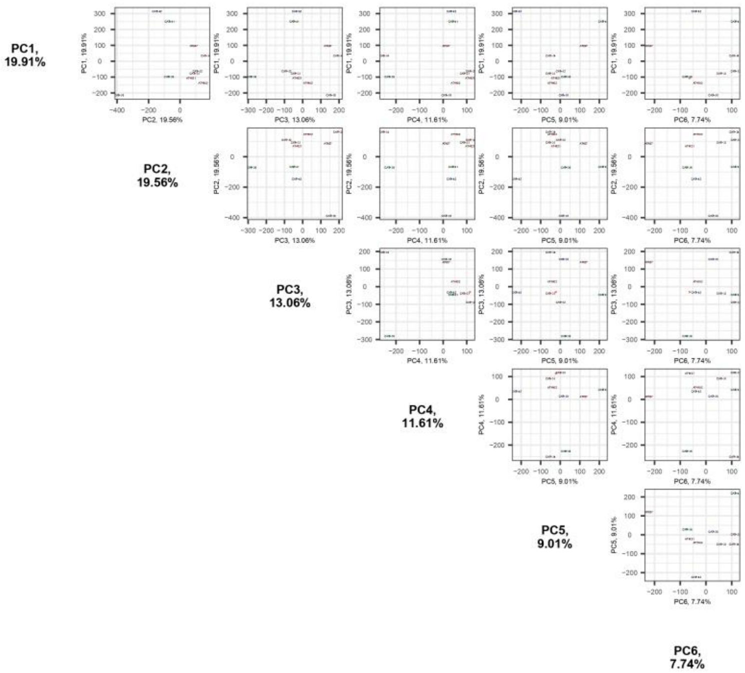

Figure S1: Scree plot that shows the cumulative variance explained by each principal component (PC).

Figure S2.

A.

Extracellular matrix organization

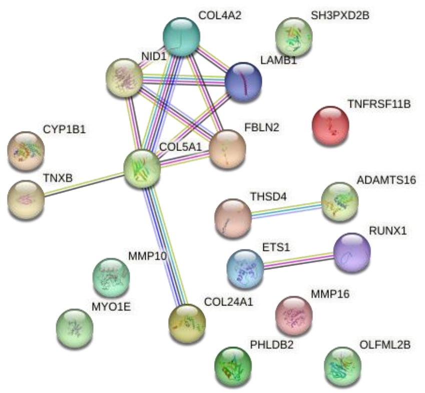

Number of nodes: 19
Average node degree: 1.37
Avg.local clustering coefficient: 0.526

Number of edges: 13
Expected number of edges: 1
PPI enrichment p-value: 3.04e-10

Cell junction assembly

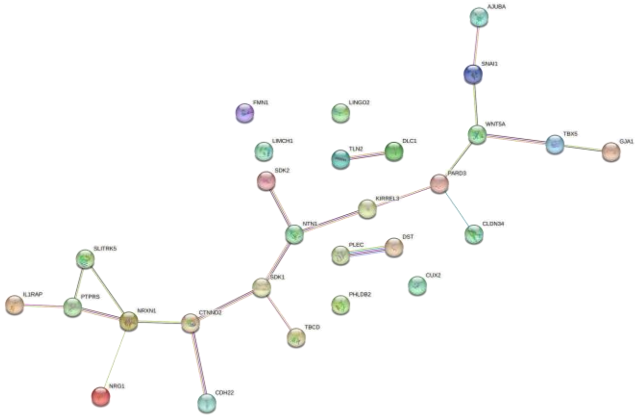

Number of nodes: 28
Average node degree: 1.5
Avg.local clustering coefficient: 0.482

Number of edges: 21
Expected number of edges: 4
PPI enrichment p-value: 3.19e-09

Cell-substrate adhesion

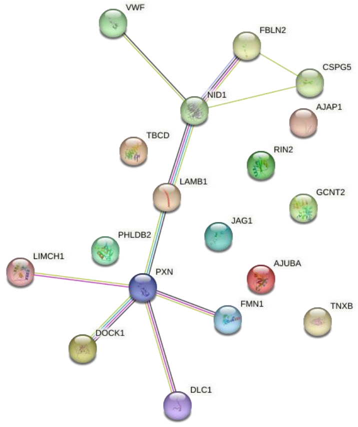

Number of nodes: 18
Average node degree: 1.11
Avg.local clustering coefficient: 0.398

Number of edges: 10
Expected number of edges: 2
PPI enrichment p-value: 1.05e-05

Figure S2: Functional protein association networks using STRING plots of the gens implicated in the extracellular matrix organization, the cell junction assembly and the cell-substrate adhesion processes
