# Supplementary material for: Smoking Suppresses the Therapeutic Potential of Adipose Stem Cells in Crohn’s Disease Patients through Epigenetic Changes
Source: Cells. 2023 Mar 27;12(7):1021. doi: 10.3390/cells12071021 (PMC10093550; doi:10.3390/cells12071021)
Supplement: Supplementary file 1 [file cells-12-01021-s001.zip › Supplementary Tables.pdf]

Supplementary Table S1. Demographic characteristics and clinical data of patients.

	Healthy	Crohn's Disease		
	**Non-smokers**	**Non-Smokers**	**Smokers**	**Ex-Smokers**
n	4	7	11	9
Disease status (Active /Inactive)	-	6/1	9/2	4/5
Age (mean ± SD)	40.5 ± 13.6	32.57 ± 6.24	37.27 ± 12.02	45.89 ± 14.38
Gender (female/male)	3/1	5/2	4/7	4/5
BMI (kg/m^2) (mean ± SD)	23.43 ± 1.5	23.41 ± 5.68	23.71 ± 3.35	24.16 ± 2.75
Biological treatment (%)	-			
None		3 (42.9)	4 (36.4)	6 (66.7)
Adalimumab		2 (28.6)	4 (36.4)	1 (11.1)
Infliximab		1 (14.3)	2 (18.2)	1 (11.1)
Ustekinumab		1 (14.3)	0 (0.0)	1 (11.1)
Vedolizumab		0 (0.0)	1 (9.1)	0 (0.0)
Age at diagnosis (%)	-			
A1		3 (42.9)	1 (10.0)	3 (33.3)
A2		4 (57.1)	7 (70.0)	2 (22.2)
A3		0 (0.0)	2 (20.0)	4 (44.4)
Location (%)	-			
L1		3 (42.9)	6 (60.0)	5 (55.6)
L2		2 (28.6)	2 (20.0)	4 (44.4)
L3		2 (28.6)	2 (20.0)	0 (0.0)
Behavior (%)	-			
B1		2 (28.6)	2 (20.0)	4 (44.4)
B2		2 (28.6)	5 (50.0)	1 (11.1)
B3		3 (42.9)	3 (30.0)	4 (44.4)
C-reactive protein (mg/dL) (median [IQR])	-	1.42 [0.80-2.30]	1.10 [0.40-13.40]	0.77 [0.61-1.00]

Abbreviations: BMI, body mass index. Age at diagnosis: A1 ≤ 16 years; A2 17–40 years; A3 > 40 years; Location:L1 = ileal; L2 = colonic; L3 = ileocolonic; Behavior: B1 = non-stenotic, non-fistulizing Crohn's disease;B2 = stenotic Crohn's disease; B3 = fistulizing Crohn's disease. a $p < 0.05$ significant differences compared with control group. Statistical test applied using R software: chisq.test() for categorical variables (with continuity correction), oneway.test() for normal continuous variables and kruskal.test() was used for the non-normal continuous variables. No significant changes were observed among groups.

Supplementary Table S2. Immunophenotypic profile of undifferentiated human adipose-derived stem cells (ASCs) isolated from non-smoker, smoker, and ex-smoker CD subjects. The values reflect the mean ±SD percentage surface positive staining of ASCs for a part of surface antigens including hematopoietic and stromal markers.

Antibody	**Non-smokers**	**Smokers**	**Ex-smokers**
**CD34**	0.15 ± 0.11	0.12 ± 0.10	0.11 ± 0.07
**CD45**	0.18 ± 0.09	0.14 ± 0.07	0.10 ± 0.08
**CD73**	97.1 ± 0.5	95.8 ± 0.9	96.6 ± 0.5
**CD90**	98.2 ± 0.9	98.1 ± 0.4	97.5 ± 0.6
**CD105**	98.9 ± 0.7	98.5 ± 0.7	99.1 ± 0.3

Supplementary Table S3. TaqMan probes used to perform qPCR

Gene symbol	Description	TaqMan probes
**IL6**	Interleukin 6	Hs00985639_m1
**TNFα**	Tumor Necrosis Factor	Hs00174128_m1
**ILB1**	Interleukin 1 Beta	Hs01555410_m1
**IL10**	Interleukin 10	Hs00961622_m1
**CD163**	CD163 Molecule	Hs00174705_m1
**MRC1**	Mannose Receptor C-Type 1	Hs00267207_m1
**CASP1**	Caspase 1	Hs00354836_m1
**TGF β**	Transforming Growth Factor Beta 1	Hs00234077_m1
**18S**	18S rRNA	Hs99999901_s1

Supplementary Table S4. Demographic characteristics and clinical data of patients used on the methylations study.

	Non-smokers	Smokers	Ex-smokers	p-value
n	5	3	2	
Disease status (%)				0.058
None (healthy)	3 (60.0)	0 (0.0)	0 (0.0)	
Active CD	2 (40.0)	2 (66.7)	0 (0.0)	
Inactive CD	0 (0.0)	1 (33.3)	2 (100.0)	
Age (mean ±SD)	43.25 ± 16.96	29.33±10.79	49±2.93	0.30
Gender (%)				0.574
F	3 (60.0)	2 (66.7)	2 (100.0)	
M	2 (40.0)	1 (33.3)	0 (0.0)	
BMI (kg/m^2) (median[IQR])	23 [21.90-24.90]	18.96 [18.48-19.43]	29.15 [26.58-31.72]	0.094
Biological treatment (%)				0.274
None	5 (100.0)	2 (66.7)	2 (100.0)	
Vedolizumab	0 (0.0)	1 (33.3)	0 (0.0)	
Immunosupresor (%)				0.127
None	4 (80.0)	1 (33.3)	0 (0.0)	
Azatioprina	1 (20.0)	2 (66.7)	2 (100.0)	
Age at diagnosis (%)				0.526
A1	0 (0.0)	1 (100.0)	1 (50.0)	
A2	2 (100.0)	0 (0.0)	1 (50.0)	
Location (%)				0.526
L1	1 (50.0)	1 (100.0)	2 (100.0)	
L3	1 (50.0)	0 (0.0)	0 (0.0)	
Behavior (%)				0.118
B2	2 (100.0)	0 (0.0)	0 (0.0)	
B3	0 (0.0)	1 (100.0)	2 (100.0)	
C-reactive protein (mg/dL) (median[IQR])	1.70[1.05-2.35]	0.18[0.14-9.74]	0.40[0.40-0.40]	0.681

Abbreviations: CD, Crohn's disease; BMI, body mass index. Age at diagnosis: A1 ≤ 16 years; A2 17–40 years; A3 > 40 years; Location: L1 = ileal; L2 = colonic; L3 = ileocolonic; Behavior: B1 = non-stenotic, non-fistulizing Crohn's disease; B2 = stenotic Crohn's disease; B3 = fistulizing Crohn's disease. Statistical test applied using R software: chisq.test() for categorical variables (with continuity correction), oneway.test() for normal continuous variables and kruskal.test() was used for the nonnormal continuous variables. No significant changes were observed among groups.

Supplementary Table S5. Top 20 hypo- and hyper-methylated genes with more of 20% of change in the comparison smokers *versus* non-smokers.

TOP 20 HYPER-METHYLATED GENES

CpG	Fold Change	pval	chr	Localization	DMSs	Gene	Biological Function
cg03720617	3,03	1,07E-06	16	Body	1	PHKB	Phosphorylase b kinase catalyzes the phosphorylation of serine in certain substrates, including troponin I.
cg13534698	2,97	1,39E-05	16	5'UTR	1	NDE1	Required for centrosome duplication and formation and function of the mitotic spindle
cg26767001	2,91	1,75E-06	12	Body	1	WNK1	Serine/threonine kinase which plays an important role in the regulation of electrolyte homeostasis, cell signaling, survival, and proliferation.
cg06560360	2,79	2,29E-05	14	Body	1	RAD51B	Involved in the homologous recombination repair (HRR) pathway of double-stranded DNA breaks arising during DNA replication or induced by DNA-damaging agents.
cg22225080	2,70	3,87E-06	18	Body	1	SETBP1	Enables histone H3K4 methyltransferase activity
cg04878315	2,57	6,69E-05	7	Body	1	HOXA9	Required for induction of E-selectin and VCAM-1, on the endothelial cells surface at sites of inflammation.
cg16533146	2,53	1,26E-06	12	Body	1	MRPS35	Help in protein synthesis within the mitochondrion
cg04891600	2,49	1,63E-07	11	TSS1500	1	CARD16	Acts as a regulator of procaspase-1/CASP1 activation implicated in the regulation of the proteolytic maturation of pro-interleukin-1 beta (IL1B) and its release during inflammation.
cg05357859	2,45	2,73E-07	6	5'UTR	1	ANKRD6	Recruits CKI-epsilon to the beta-catenin degradation complex that consists of AXN1 or AXN2 and GSK3-beta and allows efficient phosphorylation of beta-catenin, thereby inhibiting beta-catenin/Tcf signals.
cg26556684	2,37	8,75E-06	2	5'UTR	1	IL1R1	Receptor for IL1A, IL1B and IL1RN
cg25531591	2,35	5,95E-06	11	Body	1	SERGEF	Guanine nucleotide exchange factor (GEF), which may be involved in the secretion process
cg06042259	2,33	3,42E-05	7	Body	1	JAZF1	Cts as a transcriptional corepressor of orphan nuclear receptor NR2C2
cg09711028	2,28	6,62E-05	12	Body	1	ABCC9	Subunit of ATP-sensitive potassium channels (KATP)
cg14332079	2,27	1,06E-06	8	TSS1500	1	KCTD9	Substrate-specific adapter of a BCR (BTB-CUL3-RBX1) E3 ubiquitin-protein ligase complex, which mediates the ubiquitination of target proteins, leading to their degradation by the proteasome
cg14332079	2,27	1,06E-06	8	TSS1500	1	CDCA2	Regulator of chromosome structure during mitosis required for condensin-depleted chromosomes to retain their compact architecture through anaphase.
cg16311756	2,26	7,51E-06	15	5'UTR	1	CYFIP1	Component of the CYFIP1-EIF4E-FMR1 complex which binds to the mRNA cap and mediates translational repression.
cg22127684	2,25	7,06E-05	3	Body	1	GRM7	G-protein coupled receptor activated by glutamate that regulates axon outgrowth through the MAPK-cAMP-PKA signaling pathway during neuronal development
cg25068347	2,21	2,80E-06	11	Body	1	ETS1	Directly controls the expression of cytokine and chemokine genes in a wide variety of different cellular contexts
cg04498511	2,21	6,57E-05	1	5'UTR	1	ZC3H11A	RNA-binding protein that interacts with purine-rich sequences and is involved in nuclear mRNA export; probably mediated by association with the TREX complex.
cg13534901	2,17	1,61E-06	7	Body	1	AHR	Plays important roles in development, immunity and cancer

TOP 20 HYPO-METHYLATED GENES

CpG	Fold Change	pval	chr	Localization	DMS	Gene	Biological Function
cg05420790	-2,84	4,93E-07	12	1stExon	3	HOXC10	Sequence-specific transcription factor which is part of a developmental regulatory system that provides cells with specific positional identities on the anterior-posterior axis.
cg21898463	-2,52	3,55E-06	12	1stExon			
cg02009895	-2,30	1,62E-06	12	Body			
cg20340739	-2,35	4,90E-06	12	TSS1500	3	HOXC-AS3	Is a long noncoding RNA (lncRNA) that plays a crucial role in various tumors
cg05420790	-2,84	4,93E-07	12	TSS1500			
cg21898463	-2,52	3,55E-06	12	TSS1500			
cg21493516	-2,55	3,01E-06	12	5'UTR	2	HOXC4	Sequence-specific transcription factor which is part of a developmental regulatory system that provides cells with specific positional identities on the anterior-posterior axis.
cg05408649	-2,80	1,72E-06	12	5'UTR			
cg20817902	-2,28	1,90E-08	4	Body	2	PRDM8	Probable histone methyltransferase, preferentially acting on 'Lys-9' of histone H3 (By similarity).
cg23874561	-2,61	3,42E-06	4	Body			
cg17429374	-2,60	4,90E-05	11	Body	1	MACROD1	Removes ADP-ribose from aspartate and glutamate residues in proteins bearing a single ADP-ribose moiety
cg06636831	-2,50	1,05E-05	3	Body		CLEC3B	Tetranectin binds to plasminogen and to isolated kringle 4.
cg05412137	-2,02	2,26E-05	12	TSS1500	3	MIR196A2	Involved in post-transcriptional regulation of gene expression in multicellular organisms by affecting both the stability and translation of mRNAs
cg08053137	-2,49	9,39E-07	12	TSS200			
cg10662314	-2,21	6,36E-06	12	TSS1500			
cg06967452	-2,16	9,19E-05	8	Body	1	MIR1268A	Involved in post-transcriptional regulation of gene expression in multicellular organisms by affecting both the stability and translation of mRNAs
cg16663155	-2,45	1,78E-05	11	Body	1	INCENP	Component of the chromosomal passenger complex (CPC), a complex that acts as a key regulator of mitosis.
cg19274890	-2,16	5,19E-05	7	TSS200	1	DPP6	Promotes cell surface expression of the potassium channel KCND2
cg05956380	-2,11	7,35E-05	8	5ÙTR	1	SLC20A2	Role in extracellular matrix, cartilage and vascular calcification.
cg25444339	-2,09	2,52E-06	7	Body	1	HIP1	Plays a role in clathrin-mediated endocytosis and trafficking
cg18124949	-2,08	8,61E-05	3	Body	1	SCHIP1	Animal organ development; face morphogenesis; cell junction and fibroblast migration
cg12776605	-2,08	1,81E-05	3	TSS1500	1	ARPP21	Isoform 2 may act as a competitive inhibitor of calmodulin-dependent enzymes such as calcineurin in neurons.
cg04951371	-1,97	2,49E-05	2	Body	1	TSSC1	Acts as a component of endosomal retrieval machinery that is involved in protein transport from early endosomes to either recycling endosomes or the trans-Golgi network
cg15061302	-1,94	2,51E-05	3	TSS1500	2	TIRARP	ADP-ribosyltransferase that mediates mono-ADP-ribosylation of glutamate, aspartate and cysteine residues on target proteins
cg09320924	-1,92	3,70E-06	5	Body	1	SSBP2	Enables single-stranded DNA binding
cg12898442	-1,91	9,06E-05	11	3ÙTR	1	NRGN	Acts as a 'third messenger' substrate of protein kinase C-mediated molecular cascades during synaptic development and remodeling.
cg02936049	-1,89	7.46E-05	3	5ÙTR	1	ZBTB38	Transcriptional regulator with bimodal DNA-binding specificity.
cg21147635	-1.89	1.18E-05	22	5'UTR	1	MICAL3	Promotes depolymerization of F-actin by mediating oxidation of specific methionine residues on actin to form methionine-sulfoxide, resulting in actin filament disassembly and preventing repolymerization.
